# Effect of Obstructive Sleep Apnea and CPAP Treatment on Cardiovascular Outcomes in Acute Coronary Syndrome in the RICCADSA Trial

**DOI:** 10.3390/jcm9124051

**Published:** 2020-12-15

**Authors:** Yüksel Peker, Erik Thunström, Helena Glantz, Christine Eulenburg

**Affiliations:** 1 Department of Pulmonary Medicine, Koc University School of Medicine, TR 34010 Istanbul, Turkey; 2 Department of Molecular and Clinical Medicine, Sahlgrenska Academy, University of Gothenburg, SE 40530 Gothenburg, Sweden; erik.thunstrom@vgregion.se; 3 Department of Clinical Sciences, Respiratory Medicine and Allergology, Faculty of Medicine, Lund University, SE 22185 Lund, Sweden; 4 Division of Pulmonary, Allergy, and Critical Care Medicine, University of Pittsburgh School of Medicine, Pittsburgh, PA 15213, USA; 5 Department of Cardiology, Sahlgrenska University Hospital/Östra, 41345 Gothenburg, Sweden; 6 Department of Internal Medicine, Skaraborg Hospital, SE 53185 Lidköping, Sweden; helena.glantz@vgregion.se; 7 Department for Epidemiology, University of Groningen, 9712 CP Groningen, The Netherlands; christine.eulenburg@googlemail.com

**Keywords:** obstructive sleep apnea, acute coronary syndrome, cardiovascular outcomes, continuous positive airway pressure

## Abstract

We aimed to address the impact of OSA and its treatment with continuous positive airway pressure (CPAP) on major adverse cardiovascular and cerebrovascular events (MACCE) in patients with acute coronary syndrome (ACS). In this current analysis of the revascularized ACS subgroup (*n* = 353) of the Randomized Intervention with CPAP in Coronary Artery Disease and Obstructive Sleep Apnea (RICCADSA) trial (Trial Registry: ClinicalTrials.gov; No: NCT 00519597), participants with non-sleepy OSA (apnea-hypopnea-index [AHI] ≥ 15 events/h on a home sleep apnea testing, and Epworth Sleepiness Scale [ESS] score < 10; *n* = 171) were randomized to CPAP (*n* = 86) or no-CPAP (*n* = 85). The sleepy OSA patients (AHI ≥ 15 events/h and ESS ≥ 10) who were offered CPAP, and the ones with no-OSA (AHI < 5 events/h) were included in the observational arm. A post-hoc analysis was done to compare untreated OSA (no-CPAP; *n* = 78) and nonadherent sleepy/non-sleepy OSA (*n* = 96) with the reference group without OSA (*n* = 81). The primary endpoint (the first event of repeat revascularization, myocardial infarction, stroke or cardiovascular mortality) during a median 4.7-year follow-up was evaluated in time-dependent Cox proportional hazards models adjusted for confounding factors. The incidence of MACCE did not differ significantly in intention-to-treat population. On-treatment analysis showed a significant risk reduction in those who used CPAP for ≥4 vs. <4 h/day or did not receive treatment (adjusted hazard ratio [HR] 0.17; 95% confidence interval [CI] 0.03–0.81; *p* = 0.03). Compared with the reference group, nonadherent/untreated OSA was associated with an increased cardiovascular risk (adjusted HR 1.97, 95% CI 1.03–3.77; *p* = 0.04). We conclude that OSA is an independent risk factor for adverse cardiovascular outcomes in patients with ACS. CPAP treatment may reduce this risk, if the device is used at least 4 h/day.

## 1. Introduction

Acute coronary syndrome (ACS) is a term used to describe ST-elevated myocardial infarction (STEMI), or non-STEMI or unstable angina in patients with coronary artery disease (CAD). Despite advances in pharmacological therapy and revascularization procedures, ACS continues to constitute a major health burden in Western countries [1]. Obstructive sleep apnea (OSA) is overrepresented among patients with CAD [2] and previous observational cohort studies have demonstrated an independent association between OSA and major adverse cardiovascular and cerebrovascular events (MACCEs) in those patients [3,4,5].

Continuous positive airway pressure (CPAP) is the first choice of treatment for OSA, which reduces daytime sleepiness in symptomatic patients [6]. Nevertheless, most of the adults with CAD with concomitant OSA do not experience daytime sleepiness, and there is currently no consensus regarding CPAP treatment for such individuals. A previous study suggested that OSA patients who received CPAP had a reduced cardiac mortality at 5 years after percutaneous coronary intervention (PCI), compared with those who declined the treatment [7]. Other observational studies have also suggested beneficial cardiovascular effects of CPAP in clinical cohorts [8,9]. However, these findings have not been supported by the RICCADSA [10] and the SAVE [11] randomized controlled trials (RCT) in intention-to-treat (ITT) populations. More recently, the ISAACC study, not only failed to demonstrate any cardiovascular benefit of the CPAP treatment among patients with ACS but also suggested that the rate of MACCEs was not increased in the OSA group randomized to no-CPAP compared with the ACS patients without OSA [12]. Thus, there are controversies within the research field for the ACS patients after the recent results of the ISAACC trial, and there is a need for a comparative analysis to explore those associations in a similar cohort.

We have therefore hypothesized that (1) ACS patients with non-sleepy OSA randomized to CPAP would have less cardiovascular events than those not randomized to CPAP, and (2) that ACS patients with OSA that were not adherent to CPAP or assigned to no-CPAP would have an increased cardiovascular event rate compared to those without OSA.

## 2. Study Design and Methods

### 2.1. Study Participants

**Figure 1:**
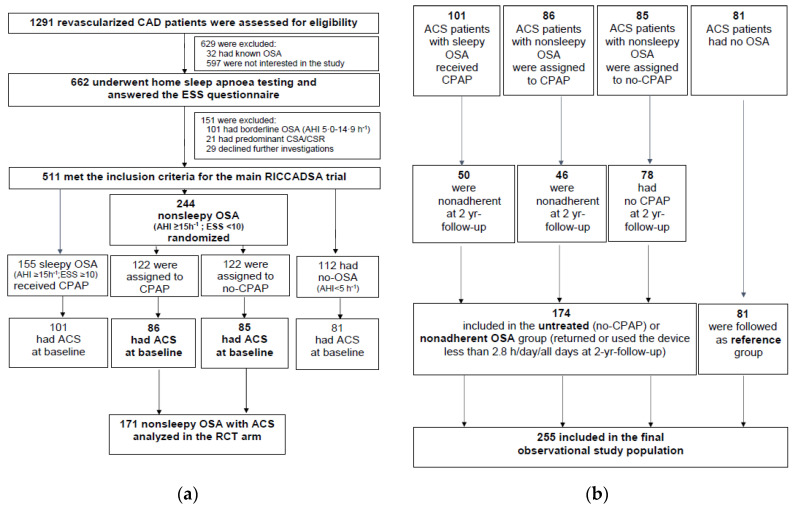
Flow of patients through the study: (**a**) The randomized controlled arm; (**b**) The final observational arm. *Abbreviations*: ACS, acute coronary syndrome; AHI, apnea-hypopnea index; CAD; coronary artery disease; CPAP, continuous positive airway pressure; CSA-CSR, central sleep apnea-Cheyne Stokes respiration; ESS, Epworth Sleepiness Scale; OSA, obstructive sleep apnea; RICCADSA, Randomized Intervention with CPAP in Coronary Artery Disease and Sleep Apnea.

The methodology of the RICCADSA trial has been published previously [13], and is detailed in the online supplement. Briefly, the study population consisted of adults with CAD, who were revascularized (PCI or coronary artery bypass grafting [CABG] surgery) in Skaraborg County (West Sweden), and had an apnea-hypopnea index (AHI) of <5/h or ≥15/h on the home sleep apnea testing (HSAT). Participants with a borderline OSA (AHI 5.0–14.9/h) and those with predominantly (more than 50%) central apneas and hypopneas of Cheyne-Stokes nature were not included (Figure 1a). The recruitment period was between December 2005 and November 2010, and the final follow-up was in May 2013. The predefined minimum follow-up was two years. CAD patients with non-sleepy OSA (AHI ≥ 15/h, Epworth Sleepiness Scale [ESS] score < 10) were randomized to CPAP or no-CPAP [10]. Participants with the sleepy OSA phenotype (AHI ≥ 15/h, ESS score ≥ 10) who were offered CPAP, and CAD patients without OSA were included in the observational arm and followed prospectively [14]. For the purpose of the current study, only patients with ACS at baseline were included in the final analysis (Figure 1).

### 2.2. Study Oversight

The Ethics Committee of the Medical Faculty of the University of Gothenburg approved the study protocol (approval no 207-05; 13 September 2005; amendment T744-10; 26 November 2010; amendment T512-11; 16 June 2011), and a written informed consent was provided from all participants. A blinded interim analysis was conducted in February 2010, and the protocol was amended for the primary endpoints of the main trial (see below). All data from hospital records and death certificates by the end of May 2013 were reviewed by an Independent Clinical Event Committee, blinded to patient identities and group allocation. The protocol and a random 10% selection of the database for baseline clinical data and follow-up procedures, including CPAP adherence and primary endpoints were monitored by a Data Monitoring Board. The trial was registered with the ClinicalTrials.gov (NCT 00519597) as well as with the national researchweb.org (FoU i Sverige—Research and development in Sweden; nr VGSKAS-4731; 29 April 2005).

### 2.3. Sleep Studies, Group Allocation and Randomization

Details of HSATs are provided in the online supplement. The 1:1 randomization of the participants with CAD and non-sleepy OSA in the main trial was scheduled with a block size of eight patients (four CPAP, four controls) stratified by sex and type of revascularization (PCI/CABG).

### 2.4. Interventions and Follow-Up

Participants with non-sleepy OSA who were randomized to CPAP were fitted with an auto-adjusting device (S8^®^ or S9^®^; ResMed, San Diego, CA, USA) by trained staff. Additional details of the follow-ups, including CPAP adherence, are given in the online supplement.

### 2.5. Statistical Analysis

For descriptive statistics, variables were reported as median and interquartile range (IQR), 25th and 75th percentile (metric variables), and as counts and percent (categorical variables). For baseline differences between the groups, the Chi-squared test and t-test were applied. The primary endpoint was the first event of MACCE (repeat revascularization, myocardial infarction, stroke and cardiovascular mortality). Each event was evaluated separately and as part of the combined endpoint. For patients who experienced more than one event during the follow-up period, only the first event was included in the combined endpoint. All-cause mortality and acute hospital admission for cardiovascular reasons were among the secondary endpoints. Criteria for the cardiovascular diagnosis defined by the ICEC are available in the online supplement.

Associations between CPAP treatment and MACCEs were tested using Cox proportional hazards models. An ITT analysis was performed comparing the patients randomized to CPAP and to no-CPAP using Cox regression. Given the fact that randomization was performed in the original CAD cohort and not in the ACS subgroup, the approach of inverse probability of treatment weighting (IPTW) was applied in the ITT analyses. The IPTW is a propensity score method that mimics randomization. Covariables age (years), body-mass-index (BMI, kg/m^2^), gender, intervention at baseline (PCI or CABG), AHI at baseline, smoking status (current smoker/nonsmoker), diabetes (yes/no), former revascularizations (yes/no), and left ventricular ejection fraction (LVEF) were included in the propensity score. Between-group differences regarding nocturnal hypoxia severity indices such as oxygen desaturation index (ODI), minimum oxygen saturation (SpO_2_) and time spent below 90% SpO_2_ at baseline were conducted but not included in the propensity score due to collinearity with the AHI. An on-treatment analysis was performed using a time-dependent Cox model to estimate the impact of CPAP usage (applying cut-off levels of averaged usage of 3, 4, and 5 h per day, respectively). With this approach, we accounted for the time-varying character of the intervention. Individual subject follow-up was split into multiple intervals according to the visit dates of the CPAP usage evaluation. Visits were planned after 1, 3, 6 and 12 months, and then annually until the end of the study. The time-dependent Cox model calculates the hazard ratios based on the individual usage intervals, with also considering the correlation of intervals belonging to the same patient.

To analyze the effect of untreated OSA, and nonadherent sleepy/non-sleepy OSA (returned or used CPAP less than 4 h/day, 70% CPAP days/period [15] corresponding 2.8 h/day/all days at 2 year-follow-up), were compared with the reference group without OSA. Univariate and multivariate Cox regressions were performed, and to be in line with the recent guidelines [16], the covariables for multivariate analyses were selected carefully. Age (years) and smoking status (yes/no) were included as well-known risk factors. Type of revascularization (PCI/CABG), diabetes (yes/no), and former intervention (yes/no) had a proven significant impact on MACCE (10), and CPAP days/period (only for the time-dependent Cox regression in the RCT arm to further characterize the usage in the on-treatment analysis) [10].

All Cox regression analyses were performed after a test for proportional hazards. All statistical tests were two-sided, and a *p*-value < 0.05 was considered significant. Statistical analysis was performed using SPSS^®^ 26.0 for Windows^®^ (SPSS Inc., Chicago, IL, USA) and Stata version 14 (StataCorp LP, College Station, TX, USA).

### 2.6. Sample Size Estimation

As previously described in detail [10], there were no studies in revascularized patients with CAD and concomitant OSA prior to 2005 to accurately inform estimates of study power for the primary outcome assessments; and a composite endpoint rate of 25% in non-sleepy patients with untreated OSA over a 3-year follow-up period was hypothesized. An interim analysis performed in February 2010 revealed an incident rate of 21%, and a CPAP adherence rate of 60% at 1 year, resulting in a protocol amendment. Thus, using a sample size of 242 patients (121 in each of the randomization arm) and an extended follow-up period of ≥2 years and ≤7 years, a significant risk reduction from 25% to 12% was hypothesized [10].

## 3. Results

### 3.1. Study Participants—The RCT Arm

As shown in Figure 1a, consecutive population of 1259 patients met the inclusion criteria for screening, of whom 662 (52.7%) agreed to participate in sleep study. A diagnostic HSAT was performed after a median of 56 days (IQR 36.5–76.0 days) following the revascularization, and participants fulfilling the inclusion criteria underwent baseline investigations after a median of 30 days (IQR 20.0–48.0 days) following the sleep recordings.

#### 3.1.1. Baseline Characteristics of the RCT Arm

A total of 171 patients with ACS and non-sleepy OSA in the RCT arm were included in the current study. Patients allocated to CPAP did not differ significantly from those allocated to no-CPAP with regard to demographic and clinical characteristics including AHI and the nocturnal hypoxia indices (Table 1).

#### 3.1.2. Numbers Analyzed

Median follow-up to the first MACCE or the end of study was 4.73 years (IQR 3.60–6.03 years). All ACS patients in the RCT arm were included in the final analysis for primary outcomes; 10 patients died (5 in CPAP group, 5 in no-CPAP group), and none was lost to follow-up. The average CPAP use was 3.53 ± 3.31 h/day at 2 year-follow-up. Of OSA patients allocated to CPAP at baseline, 34 (39.5%) returned the device within 2 years. Of the non-sleepy OSA patients allocated to no-CPAP, 3 started CPAP at baseline, and 4 during the follow-up period due to reaching the nonfatal endpoints and/or developing daytime sleepiness. CPAP compliance data at follow-ups from participants who were still using the device as well as the entire group including the patients who stopped using and returned the device are shown in Table S1 in online supplement.

#### 3.1.3. Outcomes in the RCT Arm

##### IPTW Propensity Score-Adjusted ITT Analysis

**Figure 2:**
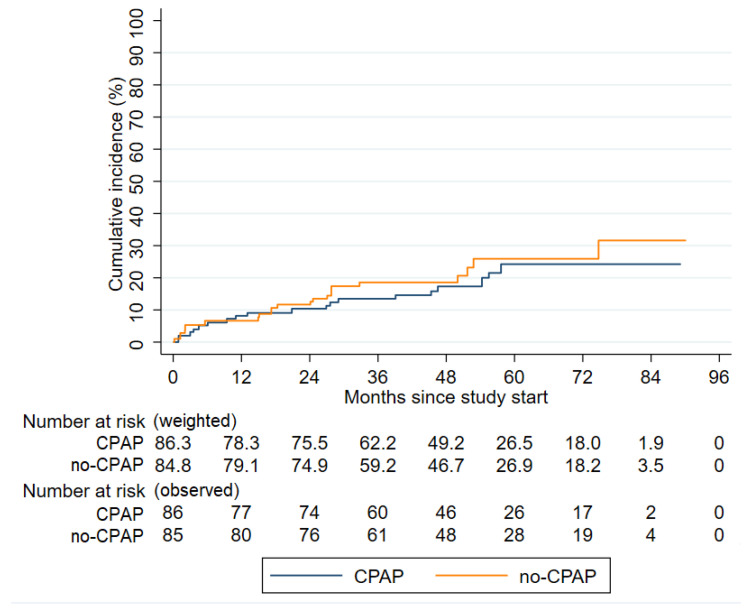
Cumulative incidences of the composite endpoint in the intention-to-treat population. CPAP, continuous positive airway pressure; OSA, obstructive sleep apnea.

In all, 37 patients reached the composite endpoint during follow-up, 19 (22.0%) in the CPAP group, and 18 (21.2%) in the no-CPAP group (not significant). The incidence of the primary endpoint did not differ significantly in non-sleepy OSA patients who did versus did not receive CPAP (5.47 vs. 5.05 per 100 person-years; hazard ratio [HR] 1.24; 95% confidence interval [CI] 0.64–2.41; *p* = 0.51). Cumulative incidences of the first MACCEs are illustrated in Figure 2.

There were no significant differences in the individual incidences of the endpoints in the PCI and CABG subgroups (see Table S2 in online supplement). On multivariate analysis, current smoking tended to be a predictor of the composite endpoint (HR 2.06; 95% CI 0.92–4.60; *p* = 0.08) whereas diabetes mellitus (HR 3.34; 95% CI 1.72–6.48; *p* < 0.001) and former revascularization (HR 3.81; 95% CI 2.03–7.14; *p* < 0.001) were significantly associated with increased risk (Table 2).

##### On-Treatment Analysis

On multivariate analysis, CPAP-usage of at least 4 h/day was associated with a significant risk reduction (adjusted HR 0.17; 95% CI 0.03–0.81; *p* = 0.03) compared with CPAP usage <4 h/day or no-CPAP (Table 3).

### 3.2. Study Participants—The Observational Arm

#### 3.2.1. Baseline Characteristics of the Observational Arm Including Nonadherent Patients

A total of 174 untreated (*n* = 78) and nonadherent (*n* = 96) OSA patients were included in the second post-hoc analysis to compare with 81 ACS patients without OSA as the reference group (Figure 1b). As shown in Table 4, OSA patients were slightly older, and had a higher rate of obesity and diabetes mellitus, and higher ESS score than ACS patients without OSA. AMI at baseline (STEMI and non-STEMI) as well as use of aspirin was more common in the no-OSA group (Table 4).

#### 3.2.2. Outcomes in the Observational Arm

**Figure 3:**
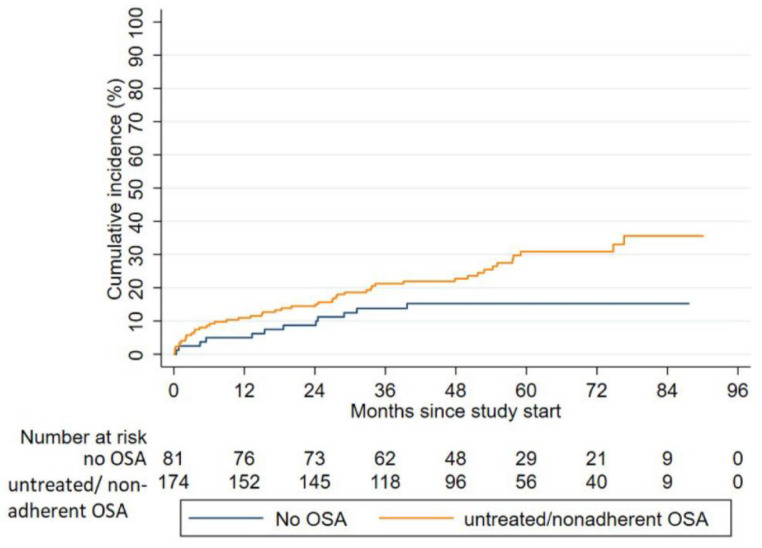
Cumulative incidences of the composite endpoint in the observational arm. OSA, obstructive sleep apnea.

Median follow-up time until death, loss to follow-up, or the end of the study was 4.9 years (IQR 3.6–6.1). The median CPAP usage was 0 (IQR 0.0–1.8 h/day/all days) at 2 year-follow-up. In all, 60 patients reached the combined endpoint during follow-up: 48 (27.6%) in the untreated/nonadherent OSA group, and 12 (14.8%) in the no-OSA group. Incidence of the first MACCE was 6.78 per 100 person-years in untreated/nonadherent OSA patients compared 3.42 per 100 person-years in the reference group without OSA (adjusted HR 1.97, 95% CI 1.03–3.77; *p* = 0.04). (Figure 3).

On multivariate analysis, diabetes mellitus tended to be a predictor of the MACCEs, and former revascularization was significantly associated with increased risk whereas CABG at baseline was protective (Table 5). There were no significant differences in the individual incidences of the endpoints in the PCI and CABG subgroups (see Table S3 in online supplement).

## 4. Discussion

This study showed that routine prescription of CPAP to adults with ACS and non-sleepy OSA had a neutral effect on the long-term cardiovascular event rate in the ITT population. A significant protective effect of CPAP was observed first after adjusting for baseline comorbidities and adherence to CPAP. Moreover, ACS patients with untreated/nonadherent OSA had an almost two-fold risk increase for MACCEs compared with the ACS patients without OSA at baseline. In addition, concomitant diabetes and previous CAD requiring revascularization seemed to be the most important conditions associated with the risk increase the current population.

The null findings in this ACS subgroup in ITT analysis confirms our previous report on the entire revascularized CAD cohort [10], and are in line with the reports from the SAVE trial [11], and more recently from the ISAAC trial [12], both with larger sample sizes than the RICCADSA population. All these three trials, conducted among adults with CVD and concomitant non-sleepy OSA, with relatively very low overall CPAP usage hours, suggest that adherence is challenging in this phenotype of patients with non-sleepy OSA. However, on-treatment analysis of the current ACS cohort also confirms our previous report on the entire CAD population, suggesting that at least 4 h of daily CPAP usage is necessary to achieve cardiovascular benefits [10]. These results were not confirmed in the SAVE and ISAACC trials using a propensity-score matched secondary analysis, which differs from the on-treatment analysis conducted in the RICCADSA trial. As discussed in a reply to a letter comparing the SAVE and RICCADSA trials’ secondary analyses, the on-treatment analysis relies on a time-dependent Cox model to estimate the association between CPAP usage and the primary composite endpoint, in which intra-individual treatment variabilities between follow-up visit intervals are considered [17]. Moreover, the comparison of untreated/nonadherent OSA patients vs. CAD patients without OSA in our study confirm the plenty of previous observational data suggesting OSA as an independent risk factor for the CVD outcomes [3,4,5,18] whereas it obviously contradicts the findings in the ISAACC trial [12].

There are multiple reasons why our results in the RCT population as well as in the post-hoc observational cohort are different from the findings in the ISAACC trial: First, the sleep studies in the ISAACC trial were conducted within 24–72 h of the hospital admission due to ACS; i.e., in a subacute setting [12] whereas the participants in the RICCADSA trial underwent HSAT in a stable clinical condition after a median of 56 days following revascularization due to the ACS. As also pointed out in a commentary [19], previous data demonstrated a high prevalence of OSA in ACS patients, but this high prevalence did not persist six months later [20]. Thus, the relationship between ACS and OSA might be bidirectional in a subacute setting, and it is therefore probable that some of the untreated OSA patients in the ISAACC trial had no-OSA at long-term follow-up. Second, almost half of the study population was current smokers at baseline in the ISAACC trial, while the proportion of smokers were around 20% at baseline in the current population (Table 1). Moreover, the severity of the ACS in terms of STEMI, non-STEMI and unstable angina as well as acute intervention with PCI with stent was poorly reported in the ISAACC trial. Thus, the high occurrence of current smoking and the comorbid conditions at baseline might have led to a ceiling effect on the adverse outcomes in the ISAACC trial. Third, the reference group, i.e., individuals without OSA, was defined as having an AHI less than 15 events per hour in the ISAACC trial while the AHI cutoff level 5 was applied for no-OSA in the RICCADSA trial, excluding the individuals with an AHI 5–15 events/h on the HSAT as a borderline group (Figure 1), who otherwise are classified as mild OSA based on full polysomnography studies [21]. A misclassification of some cases can therefore not be excluded in the ISAACC cohort [19].

Thus, the most crucial issue when evaluating the cardiovascular benefits of CPAP treatment in ACS patients with concomitant OSA without daytime sleepiness seems to be adherence to CPAP treatment. A recent meta-analysis focusing on the per protocol analysis of the RCTs, showed that adequate use of CPAP, defined as at least 4 h per day, was associated with clinically and statistically significant improvement in MACCEs [22]. In literature and clinical practice, 4 h of daily CPAP usage for 70% of the days is considered an adequate adherence to therapy, corresponding an average PAP usage of 2.8 h per day all days [15]. Of note, the downloaded CPAP usage data from the devices usually give the accumulated CPAP hours as well as CPAP hours per day, but these values refer to the days CPAP was really put on. In the current on-treatment analysis of the RCT population, we have therefore entered CPAP days/period as a covariate in the analysis, and the categorical adherence, defined as 4 h per day adjusted for CPAP days/period and baseline comorbidities, was significantly associated with reduced risk for MACCEs.

For the post-hoc observational arm, we enriched the sample of untreated OSA patients, who were allocated to no-CPAP, with nonadherent OSA patients, who returned or used CPAP less than 4 h/day, 70% CPAP days/period, corresponding 2.8 h/day/all days at 2 year-follow-up. The comparison of this untreated/nonadherent OSA group with the reference group (ACS patients without OSA) based on an AHI < 5 events/h clearly indicates that OSA is associated with increased risk for MACCEs in contrary to the findings in the ISAACC trial [12].

The strengths of this study include its randomized controlled design for patients with ACS and non-sleepy OSA with none lost to follow-up for the primary composite endpoints. Although the inclusion rate for eligible patients for HSAT screening was only 53%, the inclusion design was consecutive, and there were no significant differences in baseline characteristics of the cases undergoing versus not undergoing HSAT [10].

We should also acknowledge a number of limitations. First, the RICCADSA study was a single-center trial with two sites, which reduces generalizability of results across geographic regions. Second, the definition of “non-sleepy” OSA relied on an ESS score threshold of 10, which may not reflect an objective sleepiness in an ACS population. Nevertheless, this is a generally accepted tool for subjective daytime sleepiness, and the objective measurements such as Multiple Sleep Latency Test [23] are time consuming and not feasible to conduct in the large-scale cardiac cohorts. Third, the main RICCADSA trial was underpowered for the ITT arm for numerous reasons: CPAP adherence in CAD patients with non-sleepy OSA was lower than initially expected, which probably resulted in an inadequately powered sample size estimation. Finally, results of the on-treatment analysis as well as the post-hoc observational arm analysis should be interpreted cautiously, because no randomization to “user” versus “nonuser” is possible. Since device usage and non-usage is patient-driven, a self-selection bias can therefore not be excluded.

## 5. Conclusions

OSA is an independent risk factor for MACCEs in revascularized patients with ACS. However, routine CPAP prescription to non-sleepy OSA patients does not reduce this risk when compliance is not considered. Adequate use of CPAP, defined as at least 4 h per day, may reduce long-term adverse outcomes in ACS patients with OSA.

## Figures and Tables

**Table 1:** Demographic and clinical characteristics of the ACS patients with non-sleepy OSA randomized to CPAP versus no-CPAP at baseline *.

	CPAP *n* = 86	No-CPAP *n* = 85
Age, y	65.2 (8.4)	65.5 (8.5)
AHI, events/h	28.0 (12.2)	28.6 (13.1)
ODI, events/h	16.0 (12.8)	10.5 (10.9)
Mean SpO_2_, %	93.7 (2.4)	93.2 (1.9)
Minimum SpO_2_, %	82.4 (5.9)	82.9 (6.6)
Time spent below 90% SpO_2_, %	6.6 (16.7)	7.8 (14.2)
ESS score	5.5 (2.3)	5.3 (2.2)
BMI, kg/m^2^	28.4 (4.0)	28.7 (3.6)
LVEF, %	57.9 (8.4)	56.1 (10.3)
Obesity, %	30.2	31.8
Female, %	20.9	12.9
Current smoker, %	19.8	18.8
Pulmonary disease. %	3.5	9.4
Hypertension, %	67.4	54.1
ACS type		
STEMI, %	27.9	29.4
Non-STEMI, %	45.3	45.9
Unstable angina, %	26.7	24.7
AMI at baseline, %	73.2	75.3
CABG at baseline, %	16.3	14.1
Previous PCI or CABG, %	11.8	7.7
Diabetes mellitus, %	29.1	18.8
β-blocker use, %	91.6	86.7
Diuretic use, %	18.1	18.3
CCB use, %	22.9	14.5
ACE inhibitor use, %	55.4	50.6
ARB use, %	10.8	16.9
Clopidogrel use, %	70.2	61.4
Aspirin use, %	96.4	90.4
Warfarin use, %	3.6	7.2
Lipid-lowering agent use, %	96.4	95.2

Values are mean (SD) or percent patients. *Abbreviations*: ACE, angiotensin converting enzyme; ACS, acute coronary syndrome; AHI, apnea hypopnea index; AMI, acute myocardial infarction; ARB, angiotensin II receptor blocker; BMI, body mass index; CABG, coronary artery bypass grafting; CCB, calcium channel blocker; ESS, Epworth Sleepiness Scale; LVEF, left ventricular ejection fraction; ODI, oxygen desaturation index; OSA, obstructive sleep apnea; PCI, percutaneous coronary intervention; SD, standard deviation; SpO_2_, oxyhemoglobin saturation; STEMI, ST-elevated myocardial infarction. * None of the differences was statistically significant.

**Table 2:** Cox regression analysis of baseline covariables associated with risk for adverse cardiovascular outcomes in revascularized patients with ACS and non-sleepy OSA in the intention-to-treat analysis (*n* = 171; 37 patients reached the composite endpoint).

Multivariate	Hazard Ratio	95% CI	*p*-Value
CPAP vs. no-CPAP	1.23	0.64–2.38	0.54
Age	1.03	0.99–1.08	0.18
CABG vs. PCI	0.37	0.09–1.55	0.17
Current smoking	2.06	0.92–4.60	0.08
Diabetes mellitus	3.34	1.72–6.48	<0.01
Previous PCI or CABG	3.81	2.03–7.14	<0.01

*Abbreviations*: ACS, acute coronary syndrome; CABG, coronary artery bypass grafting; CI, confidence interval; CPAP, continuous positive airway pressure; OSA, obstructive sleep apnea; PCI, percutaneous coronary intervention.

**Table 3:** Cox regression analysis of the association between time-dependent CPAP usage (hours/day) and adverse cardiovascular outcomes in 171 revascularized patients with ACS and non-sleepy OSA. (37 patients reached the composite endpoint).

Multivariate *	Hazard Ratio	95% CI	*p*-Value
CPAP usage ≥3 h/day vs. <3 h/day or no-CPAP	1.11	0.20–6.32	0.90
CPAP usage ≥4 h/day vs. <4 h/day or no-CPAP	0.17	0.03–0.81	0.03
CPAP usage ≥5 h/day vs. <5 h/day or no-CPAP	0.26	0.07–1.05	0.06

* Adjusted for CPAP days/period, age, diabetes mellitus, current smoking, revascularization type at baseline, and former revascularization. *Abbreviations*: ACS, acute coronary syndrome; CPAP, continuous positive airway pressure; CI, confidence interval; OSA, obstructive sleep apnea.

**Table 4:** Demographic and clinical characteristics of the ACS patients with untreated or nonadherent OSA patients versus ACS patients without OSA at baseline.

	Untreated/Nonadherent OSA *n* = 174	No-OSA *n* = 81
Age *, y	64.7 (8.2)	61.4 (9.9)
AHI *, events/h	26.8 (11.8)	2.9 (1.4)
ODI *, events/h	14.4 (12.8)	1.6 (1.3)
ESS score *	7.3 (3.9)	5.5 (2.9)
BMI *, kg/m^2^	28.7 (4.0)	25.4 (2.7)
LVEF, %	56.4 (9.6)	57.7 (7.6)
Obesity, %	32.2	4.9
Female, %	11.5	19.8
Current smoker, %	22.4	32.1
Pulmonary disease. %	8.0	11.1
Hypertension, %	55.7	44.1
ACS type		
STEMI, %	27.0	33.3
Non-STEMI, %	45.4	53.1
Unstable angina, %	27.6	13.6
AMI at baseline *, %	72.4	86.4
CABG at baseline, %	16.7	11.1
Previous PCI or CABG, %	19.2	13.6
Diabetes mellitus *, %	25.9	12.3
β-Blocker use, %	89.2	82.1
Diuretic use, %	15.1	7.8
CCB use, %	15.6	9.0
ACE inhibitor use, %	53.9	46.2
ARB use, %	12.6	7.7
Clopidogrel use, %	67.5	75.3
Aspirin use, %	90.4	97.4
Warfarin use, %	7.8	2.6
Lipid-lowering agent use, %	97.0	93.5

Values are mean (SD) or percent patients. *Abbreviations*: ACE, angiotensin converting enzyme; ACS, acute coronary syndrome; AHI, apnea hypopnea index; AMI, acute myocardial infarction; ARB, angiotensin II receptor blocker; BMI, body mass index; CABG, coronary artery bypass grafting; CCB, calcium channel blocker; ESS, Epworth Sleepiness Scale; LVEF, left ventricular ejection fraction; ODI, oxygen desaturation index; OSA, obstructive sleep apnea; PCI, percutaneous coronary intervention; SD, standard deviation; STEMI, ST-elevated myocardial infarction * *p* < 0.05.

**Table 5:** Post-hoc Cox regression analysis of baseline covariables associated with risk for adverse cardiovascular outcomes in revascularized ACS patients with untreated/nonadherent OSA and no-OSA (*n* = 255; 60 patients reached the composite endpoint).

Multivariate	Hazard Ratio	95% CI	*p*-Value
Untreated/Nonadherent OSA vs. no-OSA	1.97	1.03–3.77	0.04
Age	1.02	0.98–1.05	0.33
CABG vs. PCI	0.18	0.04–0.76	0.02
Current smoking	1.51	0.82–2.77	0.18
Diabetes mellitus	1.71	0.97–3.00	0.06
Previous PCI or CABG	2.88	1.67–4.95	<0.01

*Abbreviations*: ACS, acute coronary syndrome; CABG, coronary artery bypass grafting; CI, confidence interval; CPAP, continuous positive airway pressure; OSA, obstructive sleep apnea; PCI, percutaneous coronary intervention.

## Supplementary Materials

The following are available online at https://www.mdpi.com/2077-0383/9/12/4051/s1, Supplemental Methods with References; Table S1: CPAP compliance data over time in 86 revascularized patients with acute coronary syndrome and obstructive sleep apnea; Table S2: Number of individual primary and secondary endpoint events in subgroups of the intention-to-treat population; Table S3: Number of individual primary and secondary endpoint events in subgroups of the post-hoc observational arm.
