# Supplementary material for: Effect of Obstructive Sleep Apnea and CPAP Treatment on Cardiovascular Outcomes in Acute Coronary Syndrome in the RICCADSA Trial
Source: J Clin Med. 2020 Dec 15;9(12):4051. doi: 10.3390/jcm9124051 (PMC7765306; doi:10.3390/jcm9124051)
Supplement: Supplementary file 1 [file jcm-09-04051-s001.pdf]

Methods

Study design and patients

Participants were recruited from two hospitals serving a population of approximately 250,000 living in the Skaraborg County of West Götaland, Sweden. Percutaneous coronary intervention (PCI) was performed either as an elective or acute/subacute procedure at the hospital in Skövde or at the Sahlgrenska University Hospital in Gothenburg, which was the regional hospital. Coronary artery bypass grafting (CABG) was performed in Gothenburg, and all patients were moved to the study hospitals in Skövde or Lidköping when they were clinically stable after the revascularization procedures. Eligible patients who gave informed consent to participate in the study were referred to the Sleep Medicine Unit for sleep studies. Patients with acute coronary syndrome (ACS) at baseline were included in the current substudy.

Sleep recordings

Home sleep apnea testing

The portable, home sleep apnea testing (HSAT) was performed with the Embletta® PDS (Portable Digital System) device (Embla, Broomfield, CO, USA), consisted of the following: 1) nasal pressure detector using nasal cannula/pressure transducer system, recording the square root of pressure as an index of flow; 2) thoraco-abdominal movement detection through two XactTrace™ inductive belts with respiratory inductance plethysmography (RIP) technology; 3) finger pulse oximeter detecting heart rate and oxyhemoglobin saturation (SpO_2); and 4) body position and movement detection. The patient's sleep time was estimated on the basis of self-reporting as well as the pattern of body movement during the sleep study. Patients with an estimated sleep time of <4 hours were offered a new HSAT. Apneas were defined as an almost complete (≥90%) cessation of airflow. Hypopneas were defined as a ≥50% reduction in thoracoabdominal movement and/or a ≥50% decrease in the nasal pressure amplitude for ≥10

seconds (1, 2). In addition, the total number of significant oxyhemoglobin desaturations (decrease of ≥4% from the immediately preceding baseline) were scored, and the oxygen desaturation index (ODI) was calculated as the number of significant desaturations per hour of estimated sleep. Events with a ≥30% reduction in thoracoabdominal movement and/or a ≥30% decrease in the nasal pressure amplitude for ≥10 seconds were also scored as hypopneas if there was a significant desaturation (≥4%) (2). Patients with an apnea-hypopnea index (AHI) ≥15 per hour of estimated sleep time, independent of symptom occurrence, were defined as having OSA to be included in the trial. All baseline screening recordings were scored by the same observer (YP).

Epworth Sleepiness Scale

The Epworth Sleepiness Scale (ESS) questionnaire was used to evaluate subjective excessive daytime sleepiness (3). The ESS contains eight questions to evaluate the chance of dozing off under eight scenarios in the past month. Each item is scored from 0 to 3 (0 for would never doze, 1 for slight chance of dozing, 2 for moderate chance of dozing, and 3 for high chance of dozing). The ESS score ranges from 0 to 24. Excessive daytime sleepiness was defined as an ESS score of ≥10.

Group assignment, randomization, interventions, and follow-up

Group assignment was based on the HSAT results. The 1:1 random assignment of patients with CAD and nonsleepy OSA in the main trial was scheduled by the sealed envelope system with a block size of eight patients (four CPAP, four controls) stratified by gender and revascularization type (PCI/CABG). Thus, 4 groups of sealed envelopes (8 in each group; a. PCI-men; b. PCI-women; c. CABG-men; d. CABG-women) were prepared in advance by the investigator (YP) and the study nurse. The participants were enrolled in the randomization procedure blinded to the details of the patient characteristics and comorbidity data. The patients assigned to CPAP treatment were informed about the technical procedure and provided with an auto-titrating CPAP

device (S8®, or S9®; ResMed, Sydney, Australia) and a nasal or full-face mask and humidifier by trained staff at the study center. All participants assigned to CPAP were instructed to use the device at home every night for ≥4 hours, contacted by telephone after one week and given a check-up in the clinic after 1 month, 3 months, 6 months, 1 year, and then yearly to end of the main study. All OSA patients who were obese were given advice about weight reduction. All participants were evaluated at 3, 6, and 12 months, and annually thereafter, and were given usual care by their physicians. A new HSAT was performed in all patients at 3 and 12 months, and annually thereafter (with CPAP in treated OSA patients) as part of a planned future post-hoc analysis comparing reports from the CPAP devices regarding residual AHI and pressures applied during CPAP treatment, and for analysis of the natural course of OSA in patients who were randomized to no-CPAP.

Adherence to CPAP

OSA patients receiving CPAP treatment brought their device to the clinic at each scheduled follow-up visit; monitoring settings and hours of CPAP use were obtained from the machines' internal clocks and recorded. In addition, pressure level, mask leak and residual AHI measures were noted. All necessary adjustments of the CPAP device and mask fittings were done according to clinical routines by the sleep medicine unit staff. Patients who were unable to adhere to CPAP treatment were followed as part of the treatment arm as defined in the intention-to-treat analysis.

Cardiovascular endpoint criteria

An Independent Clinical Event Committee (ICEC) reviewed all data obtained from hospital records and death certificates by the end of May 2013, blinded to personal identity and group allocation. The ICEC review was based on a previously described definition of the endpoints (4), which was applied in the HOT study (5), and other trials. In summary, overall mortality was

based on the death certificate. Cardiovascular mortality was defined as death from any of the following: myocardial infarction, stroke (cerebral haemorrhage or cerebral infarction), ruptured aortic aneurysm (thoracic or abdominal), heart failure (as determined by the treating physician), sudden death with no cause other than presumed cardiac (malignant arrhythmias), death during or within 28 days of CABG or PCI, and pulmonary embolism. Myocardial infarction was defined as ≥2 of the following signs/symptoms: sudden chest pain and/or sudden shortness of breath and/or syncope; new left bundle branch block or new ST-elevation or transient ST- or T-wave changes; increase of troponin I levels to >0.10 µg/L in ≥2 samples or increases in myocardial necrosis biomarkers (other causes of troponin elevation should be excluded). Evidence of myocardial infarction at autopsy could also be used as a single criterion. Stroke was defined as sudden onset of focal neurological signs lasting >24 hours (other causes such as brain tumor, subdural or epidural hematoma, subarachnoid haemorrhage, psychosomatic, peripheral nerve lesions should be excluded). Stroke was defined as cerebral hemorrhage if computed tomography (CT) or magnetic resonance imaging (MRI) of the brain showed intracerebral blood, and as cerebral infarction if early CT brain was normal and subsequent follow-up was compatible with stroke, or if later CT brain or MRI showed signs of infarction; or, as a single criterion, evidence of cerebral haemorrhage or infarction at autopsy and determined by the pathologist as the cause of death. CABG was defined as an operation with grafts to coronary arteries, and PCI was defined as dilatation of the coronary arteries with or without stents. Pulmonary embolism was defined sudden onset of chest pain and/or shortness of breath and/or syncope together with typical CT findings of the pulmonary arteries or pulmonary scintigraphy. Aortic aneurysm (either thoracic or abdominal) was defined as all 3 of: sudden onset of chest pain or abdominal pain; typical findings on chest or abdominal radiography or ultrasound; need for intervention (blood pressure treatment, or surgery, or percutaneous transluminal intervention with or without stent). Acute hospital

admissions for cardiovascular reasons included myocardial infarction, stroke, pulmonary embolism, aortic aneurysm (as defined above) as well as acute hospital admissions for heart failure, transient ischemic attacks, chest pain of presumed cardiac origin (e.g. angina pectoris), peripheral emboli, atrial fibrillation and other cardiac arrhythmias, and intermittent claudication.

Data collection and analysis

The primary outcome variables were documented prospectively and were not subject to observer bias. Baseline comorbidity data, results of sleep recordings, and CPAP compliance data were prospectively recorded in separate files at a specific server of the study hospital by research personnel blinded to study group allocation and/or unaware of the study outcomes.

REFERENCES

1. Norman RG, Ahmed MM, Walsleben JA, Rapoport DM. Detection of respiratory events during NPSG: nasal cannula/pressure sensor versus thermistor. *Sleep* 1997; 20: 1175-1184.
2. Sleep-related breathing disorders in adults: recommendations for syndrome definition and measurement techniques in clinical research. The Report of an American Academy of Sleep Medicine Task Force. *Sleep* 1999; 22: 667-689.
3. Johns MW. A new method for measuring daytime sleepiness: the Epworth sleepiness scale. *Sleep* 1991; 14: 540-545.
4. Hansson L, Hedner T, Dahlöf B. Prospective randomized open blinded end-point (PROBE) study. A novel design for intervention trials. Prospective Randomized Open Blinded End-Point. *Blood Press* 1992; 1: 113-119.
5. Hansson L, Zanchetti A, Carruthers SG, Dahlöf B, Elmfeldt D, Julius S, Ménard J, Rahn KH, Wedel H, Westerling S. Effects of intensive blood-pressure lowering and low-dose aspirin in patients with hypertension: principal results of the Hypertension Optimal Treatment (HOT) randomised trial. HOT Study Group. *Lancet* 1998; 351: 1755-1762.

Table S1. CPAP compliance data over time in 86 revascularized patients with acute coronary syndrome and obstructive sleep apnea (patients who stopped using the devices are excluded)

	CPAP devices checked	CPAP use (hours/day)	CPAP usage (days/period)	95th centile (cmH_2O)	Residual AHI (events/hour)
At 1 month	71	4·4 (2·3)	68·9 (30·1)	9·3 (2·1)	6·2 (5·7)
At 3 months	64	5·0 (2·2)	71·1 (27·3)	9·7 (1·6)	6·3 (5·0)
At 6 months	59	5·3 (2·0)	69·9 (29·1)	9·5 (1·7)	5·6 (3·7)
At 1 year	55	5·8 (1·7)	75·9 (24·6)	9·6 (1·5)	5·7 (3·7)
At 2 years	49	6·1 (1·8)	73·3 (25·2)	9·6 (2·6)	5·7 (3·8)
At 3 years	38	6·3 (1·7)	73·9 (23·1)	9·5 (1·6)	6·2 (3·6)
At 4 years	22	6·4 (1·6)	73·5 (21·4)	8·8 (1·8)	5·4 (3·1)
At 5 years	13	6·7 (1·0)	71·5 (17·5)	11·1 (4·7)	4·9 (3·6)
At 6 years	6	7·1 (0·7)	63·3 (24·3)	10·1 (2·3)	5·1 (2·9)

Values are mean (standard deviation).

Definition of abbreviations: AHI, apnea hypopnea index; CPAP, continuous positive airway pressure.

Table S2. Number of individual primary and secondary endpoint events in subgroups of the intention-to-treat population (between-group differences were not statistically significant).

	**Nonsleepy OSA on CPAP**	**Nonsleepy OSA no-CPAP**
Overall	n=86	n=85
Repeat revascularization	15	9
Acute myocardial infarction	10	6
Stroke	3	4
Cardiovascular death	2	4
Noncardiovascular death	3	1
Acute hospital admissions for CVD	24	23
PCI subgroup	n=57	n=64
Repeat revascularization	15	9
Acute myocardial infarction	9	6
Stroke	2	4
Cardiovascular death	1	4
Noncardiovascular death	2	1
Acute hospital admissions for CVD	21	23
CABG subgroup	n=14	n=12
Repeat revascularization	0	0
Acute myocardial infarction	1	0
Stroke	1	0
Cardiovascular death	1	0

Noncardiovascular death	1	0
Acute hospital admissions for CVD	3	0

Definition of abbreviations: CABG, coronary artery bypass grafting; CVD, cardiovascular disease; CPAP, continuous positive airway pressure; OSA, obstructive sleep apnea; PCI, percutaneous coronary intervention.

Table S3. Number of individual primary and secondary endpoint events in subgroups of the post-hoc observational arm (between-group differences were not statistically significant).

	**Untreated/Nonadherent OSA**	**No-OSA**
Overall	n=174	n=81
Repeat revascularization	33	10
Acute myocardial infarction	21	6
Stroke	7	1
Cardiovascular death	7	2
Noncardiovascular death	5	1
Acute hospital admissions for CVD	56	20
PCI subgroup	n=145	n=72
Repeat revascularization	33	9
Acute myocardial infarction	20	6
Stroke	7	1
Cardiovascular death	7	2
Noncardiovascular death	2	1
Acute hospital admissions for CVD	52	19
CABG subgroup	n=29	n=9
Repeat revascularization	0	1

Acute myocardial infarction	1	0
Stroke	0	0
Cardiovascular death	0	0
Noncardiovascular death	3	0
Acute hospital admissions for CVD	4	1

Definition of abbreviations: CABG, coronary artery bypass grafting; CVD, cardiovascular disease; OSA, obstructive sleep apnea; PCI, percutaneous coronary intervention.
